# Association between the unaccompanied nursing care model and postoperative delirium in older adults with hip fractures: a retrospective before-and-after cohort study

**DOI:** 10.3389/fmed.2026.1850834

**Published:** 2026-07-06

**Authors:** Cuitian He, Qian Liao, Qi Tan, Baitao Yang, Zaoxiong Wen, Yitao Yao, Zixing Zhao

**Affiliations:** 1 Department of Joint Surgery and Sports Medicine, The Sixth People's Hospital of Huizhou, Affiliated Huiyang Hospital of Southern Medical University, Huizhou, China; 2 Department of General Practice, Danshui Street Second Community Health Service Center of Huiyang District, Huizhou, Guangdong Province, China

**Keywords:** hip fractures, older adults, postoperative delirium, traditional accompanied nursing care model, unaccompanied nursing care model

## Abstract

**Background:**

Reduced family participation and restricted visitation have been shown to increase the risk of delirium in older adults. The unaccompanied nursing care model (UNCM), characterized by limited family participation and reduced visitation frequency, differs from the traditional accompanied nursing care model (TNCM), which allows greater family participation. However, clear evidence is lacking regarding whether UNCM, compared with TNCM, is associated with an increased incidence of postoperative delirium (POD) in older adults with hip fractures. Therefore, this study aimed to investigate the potential association between UNCM and POD in this population.

**Methods:**

This retrospective cohort study analyzed the medical records of patients who underwent hip fractures surgery at a tertiary medical center between January 2022 and December 2025. Patients were divided into TNCM and UNCM groups according to the UNCM implementation date at this institution (1 July 2024). Propensity score matching (PSM) was performed to balance baseline characteristics between the groups. A 1.59:1 matching approach with a caliper width of 0.2 was used to reduce confounding bias. Covariate balance was evaluated using standardized mean differences (SMDs). A multivariable logistic regression analysis was conducted to examine the independent association between UNCM and POD. Subgroup analyses and interaction tests were performed to explore the potential effect of modification.

**Results:**

A total of 645 patients were enrolled, with an overall POD incidence of 14.42%. The POD incidence was 13.9% in the TNCM group and 15.29% in the UNCM group, with no significant difference between the groups (*P* = 0.626). A multivariable logistic regression analysis showed no statistically significant difference in POD incidence between the two nursing care models [odds ratio (OR) = 1.24, 95% confidence interval (CI): 0.75–2.06, *P* = 0.395]. Covariate balance was achieved after PSM, and the results remained unchanged (OR = 0.88, 95% CI: 0.52–1.46, *P* = 0.613). Subgroup analyses showed no significant interaction effects across the stratified variables (all *P-values* for interaction > 0.05).

**Conclusion:**

UNCM may not increase the occurrence of POD in older adults with hip fractures and limited family participation.

## 1 Introduction

Postoperative delirium (POD) is one of the most common and severe neurocognitive disorders among older adults with hip fractures, significantly increasing mortality, prolonging hospital stay, and imposing substantial burdens on society and families (1, 2). Its pathophysiological mechanism is complex and has not been fully elucidated. POD is currently considered to result from the combined effects of multiple factors, including advanced age, preoperative dementia, malnutrition, frailty, anesthesia type, postoperative pain, inflammatory response, and anemia (3, 4). Family participation is a key component of non-pharmacological interventions for POD prevention (5). Several studies have reported that restricted family visitation is associated with an increased incidence of POD (6, 7).

The traditional accompanied nursing care model (TNCM), which relies on family members as primary caregivers, faces multiple challenges, including the growing number of empty-nest older adults, shrinking family size, labor migration, and increasing demand for professional nursing care (8). In this context, the unaccompanied nursing care model (UNCM) has emerged (9). In China's healthcare system, UNCM generally refers to a comprehensive and continuous nursing care model that provides comprehensive life support and medical care through professional caregivers arranged by hospitals or third-party institutions. This model aims to standardize nursing practices, improve nursing care quality, and alleviate the burden on families (10). This new nursing care model, characterized by family absence, fundamentally alters patients' social support and interpersonal environment during hospitalization.

Nevertheless, the association between UNCM and POD risk in older adults with hip fractures remains unclear. High-quality clinical studies, particularly randomized controlled trials and systematic reviews directly comparing the effects of UNCM and TNCM on the incidence of POD in older adults with hip fractures, are lacking. This limits the standardized application and promotion of UNCM in clinical practice. Therefore, this single-center, large-scale, retrospective cohort study was designed to investigate the association between UNCM and POD in older adults with hip fractures and to provide evidence for optimizing perioperative care strategies and improving clinical outcomes in this population.

## 2 Methods

### 2.1 Study design and ethical considerations

This was a single-center, retrospective, before-and-after cohort study. Clinical data were collected from older adults with hip fractures who underwent surgical treatment at our hospital between 1 January 2022 and 31 December 2025. Perioperative management during this period was performed by the same medical and nursing teams. Patients were divided into two groups according to the UNCM implementation date (1 July 2024). Patients enrolled before these dates were classified as the TNCM group, and those enrolled after these dates were classified as the UNCM group. The study protocol was approved by the Institutional Review Board of our institution. Because this retrospective study involved only the analysis of clinical medical records without direct patient intervention or disclosure of identifiable information, informed consent was waived. During data collection and analysis, all personal identifying information, including names, hospital record numbers, and identity card numbers, was strictly encrypted and anonymized to protect patient privacy. This study was conducted in accordance with the ethical principles of the Declaration of Helsinki.

### 2.2 Study population

All data were collected retrospectively by reviewing the Hospital Information System, Electronic Medical Record System, and Nursing Document Recording System. Data were independently extracted by two uniformly trained researchers, and the results were verified. Discrepancies were resolved by a third senior researcher to ensure data quality. The inclusion criteria were as follows: (1) age ≥ 65 years and (2) fragile hip fractures (including femoral neck and intertrochanteric fractures) treated surgically. The exclusion criteria were as follows: (1) multiple trauma or fractures, (2) transfer to the intensive care unit, (3) pre-existing neurological or psychiatric disorders, (4) terminal-stage malignant tumors, (5) government relief recipients without family members, (6) hospitalization spanning both time intervals, and (7) incomplete electronic medical records.

### 2.3 Exposure

The UNCM policy was implemented at our institution on 1 July 2024. Before that date, all hospitalized patients, except government relief recipients, were cared for by family members or self-employed private caregivers. Family members played a dominant role, with no restrictions on the duration or frequency of visits. These patients were classified as receiving TNCM. After implementation, hospitalized patients were cared for by professional caregivers provided by third-party institutions. Family visits were strictly restricted in duration and frequency, allowed only between 10:00 and 13:00 and between 16:00 and 19:00. These patients were classified as receiving UNCM.

### 2.4 Outcome

The main outcome was POD, occurring from the first postoperative day to discharge. The diagnosis of delirium was based on neurological consultation records or a confirmed clinician diagnosis in accordance with the Diagnostic and Statistical Manual of Mental Disorders, Fifth Edition (DSM-5) criteria. For cases without a documented formal diagnosis, two researchers retrospectively assessed delirium using the Confusion Assessment Method (CAM) by reviewing data from the Electronic Medical Record System and Nursing Document Recording System. Disagreements were adjudicated by a senior clinician.

We adopted standardized screening criteria to identify potential POD cases based on descriptive terms documented in the medical records. The inclusion criteria were as follows: (1) postoperative medical records containing “mental status change,” “confusion,” “disorientation,” “agitation,” “delirium,” “inappropriate behavior,” “inattention,” “hallucinations,” and “combative behavior”; and (2) postoperative drug regimens containing “quetiapine,” “olanzapine,” “haloperidol,” or “risperidone.” The exclusion criteria were as follows: (1) preoperative medical records containing the symptoms listed above and (2) preoperative drug regimens containing the drugs listed above. The CAM consists of four key diagnostic criteria: (1) acute onset of symptoms with a fluctuating clinical course, (2) inattention, (3) disorganized thinking, and (4) altered level of consciousness. A confirmed diagnosis of POD required both criteria 1 and 2, plus either criterion 3 or 4.

### 2.5 Covariates

To control for potential confounding factors, the following baseline characteristics and perioperative variables were collected. These variables were selected based on known risk factors for POD reported in relevant literature and included gender, age, fracture type, time to surgery, operative duration, intraoperative blood loss, blood transfusion status, American Society of Anesthesiologists (ASA) classification, anesthesia method, leukocyte count, platelet count, hemoglobin level, creatinine level, albumin level, and comorbidities such as diabetes mellitus, cardiovascular disease, cerebrovascular disease, chronic pulmonary disease, and renal insufficiency.

### 2.6 Statistical analysis

Descriptive statistics were used to summarize patients' baseline characteristics. Continuous variables were expressed as mean ± SD and compared using the independent-samples *t*-test if normally distributed; non-normally distributed variables were expressed as median (IQR) and analyzed using the Mann–Whitney U-test. Categorical variables are reported as *n* (%) and compared using the chi-squared test or Fisher's exact test.

A univariate logistic regression analysis was performed to examine the primary associations between the nursing care model, collected covariates, and POD; the results are shown as odds ratios (ORs) and 95% confidence intervals (CIs). Subsequently, a multivariate logistic regression model was constructed with the nursing care model as the independent variable, and variables with a *P-value* of < 0.10 in the univariate analysis were included in the model. Adjusted ORs and 95% CIs were reported. The goodness of fit of the model was assessed using the Hosmer–Lemeshow test.

To minimize selection bias and imbalance in baseline characteristics caused by non-random grouping, propensity score matching (PSM) was performed. The nursing care model was used as the dependent variable, and a 1.59:1 nearest-neighbor matching method was applied with a caliper width of 0.2. Standardized mean differences (SMDs) were calculated for all covariates between the two groups and compared before and after matching. An SMD of < 0.10 was considered to indicate a satisfactory balance between groups.

To explore whether the association between the nursing care model and POD varied across subgroups, the multivariable logistic regression analysis was repeated within each subgroup, and the significance of potential interactions was tested.

All statistical analyses were performed using R software (version 4.3.0). All tests were two-sided, and statistical significance was set at a *P-value* of < 0.05.

## 3 Results

### 3.1 Study characteristics

**Figure 1:**
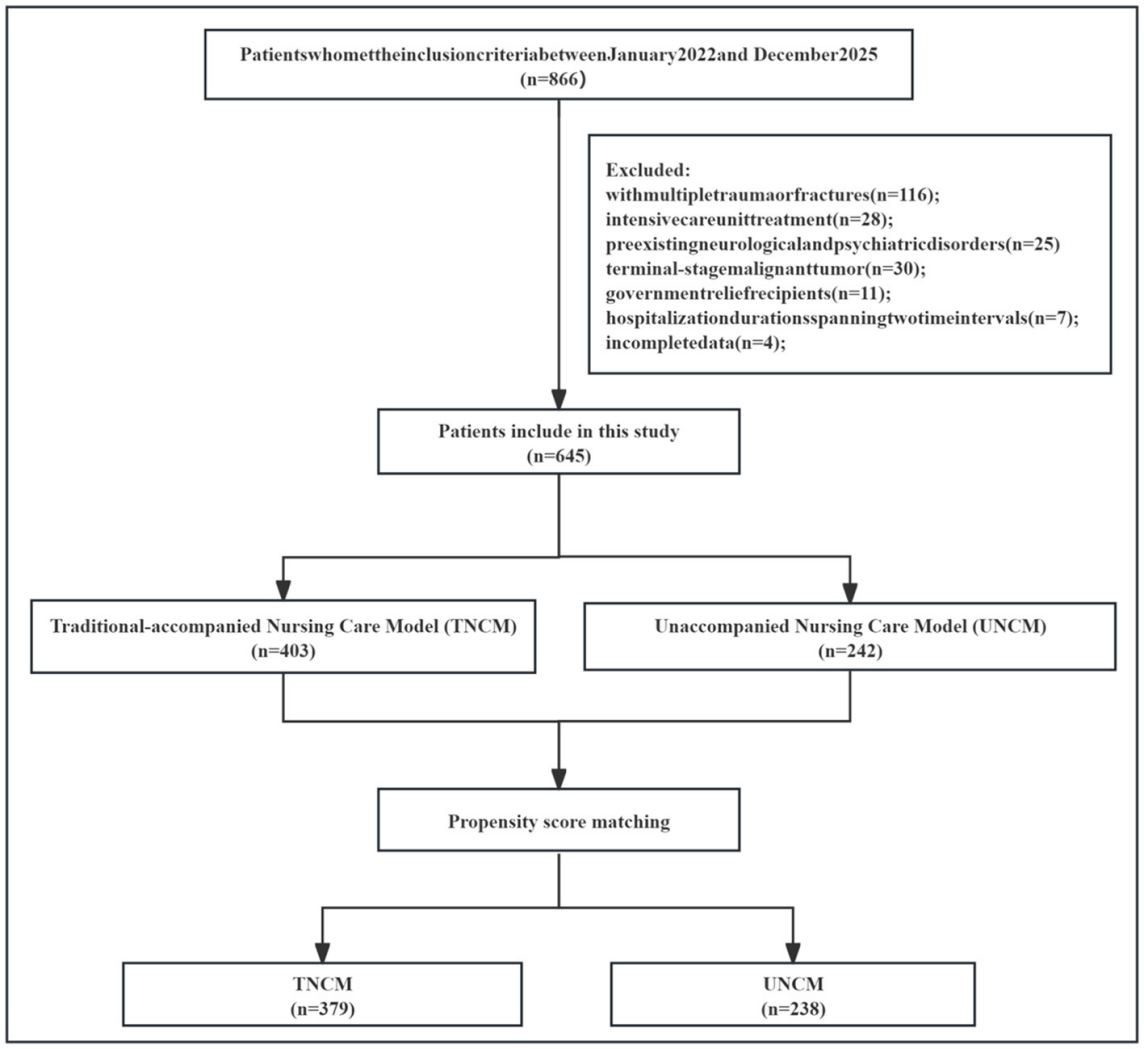
Flowchart of the study design.

A total of 866 older adults with hip fractures who underwent surgery met the inclusion criteria. After 221 patients were excluded, including 116 with multiple trauma or multiple fractures, 28 who were transferred to the intensive care unit, 25 with pre-existing neurological or psychiatric disorders, 30 with terminal-stage malignant tumors, 11 government relief recipients, 7 whose hospitalization spanned both time intervals, and 4 with incomplete electronic medical records, 645 patients were enrolled. The patient selection flowchart is shown in Figure 1.

Among the 645 patients, 458 were female (71.01%), and the mean age was 82 years. There were 311 femoral neck fractures (48.22%) and 334 intertrochanteric fractures (51.78%). The mean time to surgery was 5 days. Comorbidities included diabetes mellitus in 152 patients (23.57%), cardiovascular disease in 397 patients (61.55%), cerebrovascular disease in 243 patients (37.67%), chronic pulmonary disease in 207 patients (32.09%), and renal insufficiency in 76 patients (11.78%). A majority of baseline characteristics were comparable between the groups (*P* > 0.05), except for time to surgery (*P* = 0.038) and surgery duration (*P* = 0.034). After PSM, all baseline characteristics were comparable between the groups (*P* > 0.05). Detailed baseline characteristics are shown in Supplementary Table 1.

### 3.2 Propensity score matching analysis

**Figure 2:**
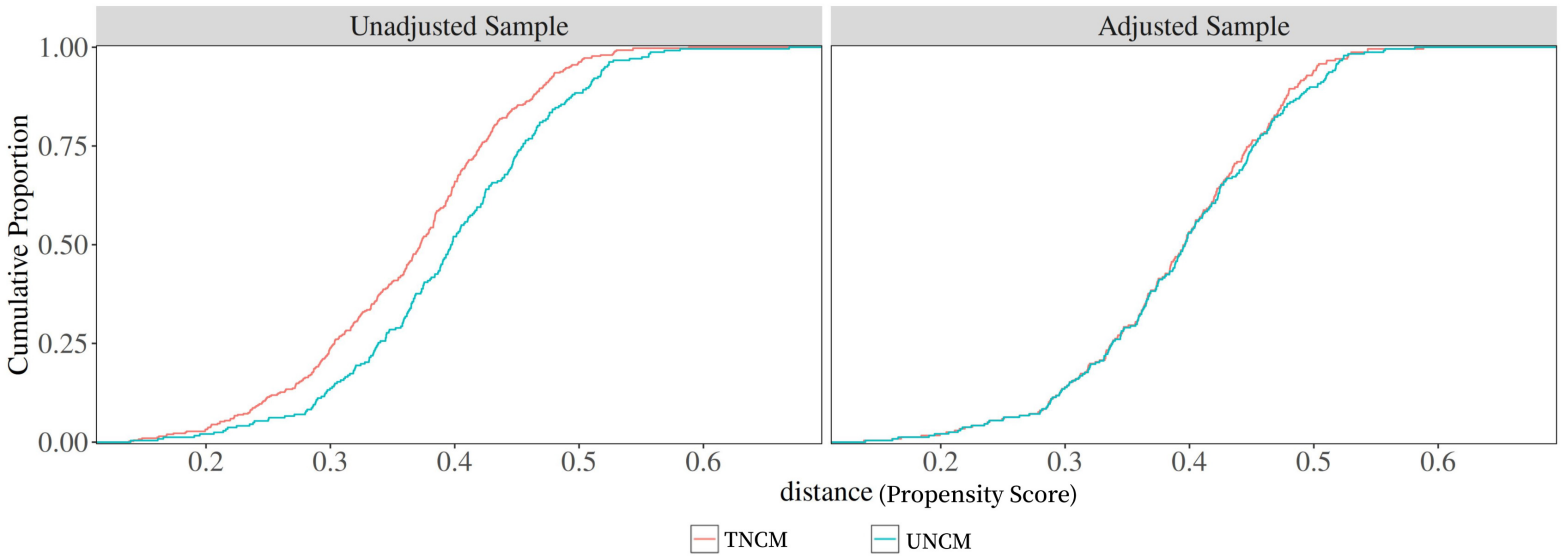
Propensity score density plot before and after PSM.

A PSM cohort was established by matching nine key covariates: gender, age, time to surgery, cerebrovascular disease, surgery duration, blood loss, blood transfusion, ASA classification, and anesthesia method. We performed 2:1 nearest-neighbor matching without replacement with a caliper width of 0.2 standard deviations of the propensity score. Because of sample size and common support restrictions, the final matched ratio was approximately 1.59:1. Overall, 379 patients in the TNCM group were matched with 238 patients in the UNCM group. After matching, the imbalance in baseline characteristics was significantly reduced, and SMDs for most variables were less than 0.10, indicating a good balance between the groups. The baseline characteristics of the two groups are presented in Supplementary Table 1. The propensity score density plot further supported this finding (Figure 2): Propensity score distributions differed substantially between the groups before matching (Figure 2, left), whereas the distributions overlapped substantially after matching (Figure 2, right). This suggests that the baseline confounding bias was substantially reduced. Before matching, the overall POD incidence was 14.42%, with no significant difference between the TNCM (13.90%) and UNCM (15.29%) groups (*P* = 0.626). This trend remained unchanged after matching: The overall incidence of POD was 14.26%, with 13.98% in the TNCM group and 14.71% in the UNCM group (*P* = 0.803). The incidence rates of POD are shown in Table 1.

**Table 1:** POD incidence in the study population.

Variable	Unadjusted					Adjusted				
	Total (*n* = 645)	TNCM (*n* = 403)	UNCM (*n* = 242)	*P*	SMD	Total (*n* = 617)	TNCM (*n* = 379)	UNCM (*n* = 238)	*P*	SMD
POD, *n* (%)				0.626					0.803	
No	552 (85.58)	347 (86.10)	205 (84.71)		−0.039	529 (85.74)	326 (86.02)	203 (85.29)		−0.020
Yes	93 (14.42)	56 (13.90)	37 (15.29)		0.039	88 (14.26)	53 (13.98)	35 (14.71)		0.020

TNCM, traditional-accompanied nursing care model; UNCM, unaccompanied nursing care model; SMDs, standardized mean differences.

### 3.3 Univariate and multivariable logistic regression analyses

Univariate and multivariable logistic regression analyses were performed to investigate associations between variables and POD before and after matching. Before matching, eight variables were significantly associated with POD in the univariate logistic regression analysis (*P* < 0.05). After adjusting for potential confounders in the multivariate logistic regression analysis, four variables were identified as independent risk factors for POD (*P* < 0.05): age (OR = 1.06, 95% CI: 1.02–1.10), fracture type (OR = 0.39, 95% CI: 0.23–0.68), leukocyte count (OR = 0.90, 95% CI: 0.83–0.97), and cerebrovascular disease (OR = 2.78, 95% CI: 1.63–4.74). Univariate and multivariable analyses before PSM are presented in Table 2. After matching, six variables were significantly associated with POD in univariate logistic regression analysis (*P* < 0.05). Five variables were identified as independent risk factors for POD in multivariate logistic regression analysis (*P* < 0.05): age (OR = 0.95, 95% CI: 0.92–0.99), fracture type (OR = 2.89, 95% CI: 1.64–5.10), leukocyte count (OR = 1.11, 95% CI: 1.02–1.20), cerebrovascular disease (OR = 0.37, 95% CI: 0.22–0.65), and renal insufficiency (OR = 0.51, 95% CI: 0.27–0.98). Univariate and multivariate analyses after PSM are shown in Table 3.

**Table 2:** Univariate and multivariate analyses of POD risk factors before PSM.

Variables	Univariate		Multivariate	
	OR (95% CI)	*P*	OR (95% CI)	*P*
Nursing care model	1.12 (0.71–1.75)	0.626	1.24 (0.75–2.06)	0.395
Gender	0.74 (0.47–1.19)	0.214	0.88 (0.52–1.49)	0.626
Age	1.07 (1.04–1.11)	**< 0.001**	1.06 (1.02–1.10)	**0.002**
Time to surgery	1.03 (0.99–1.07)	0.108	1.04 (0.99–1.09)	0.106
Fracture type	0.39 (0.24–0.62)	**< 0.001**	0.39 (0.23–0.68)	**< 0.001**
Surgery duration	1.00 (1.00–1.01)	0.233	1.00 (1.00–1.01)	0.212
Blood loss	1.00 (1.00–1.00)	0.447	1.00 (1.00–1.00)	0.299
Blood transfusion	1.24 (0.67–2.31)	0.497	0.85 (0.36–2.03)	0.717
ASA	2.76 (1.43–5.32)	**0.002**	1.41 (0.68–2.92)	0.361
Anesthesia method	1.13 (0.49–2.62)	0.771	1.36 (0.53–3.52)	0.527
Leukocyte	0.89 (0.83–0.96)	**0.002**	0.90 (0.83–0.97)	**0.007**
Platelet	1.00 (1.00–1.00)	0.593	1.00 (1.00–1.00)	0.735
Hemoglobin	1.00 (0.98–1.03)	0.643	1.00 (0.98–1.02)	0.931
Creatinine	1.00 (0.99–1.00)	0.266	1.00 (0.99–1.00)	0.335
Albumin	1.02 (0.97–1.07)	0.392	1.01 (0.96–1.07)	0.599
Diabetes	1.15 (0.70–1.91)	0.582	1.12 (0.61–2.06)	0.707
Cardiovascular	1.73 (1.07–2.81)	**0.026**	1.43 (0.83–2.47)	0.2
Cerebrovascular	2.80 (1.79–4.39)	**< 0.001**	2.78 (1.63–4.74)	**< 0.001**
Chronic pulmonary	2.64 (1.69–4.12)	**< 0.001**	1.41 (0.83–2.40)	0.204
Renal insufficiency	2.86 (1.64–4.98)	**< 0.001**	1.86 (0.98–3.53)	0.059

OR, odds ratio; CI, confidence interval; ASA, American Society of Anesthesiologists; Bold values indicate statistical significance (*p* < 0.05).

**Table 3:** Univariate and multivariate analyses of POD risk factors after PSM.

Variables	Univariate		Multivariate	
	OR (95% CI)	*P*	OR (95% CI)	*P*
Nursing care model	0.94 (0.59–1.50)	0.803	0.88 (0.52–1.46)	0.613
Gender	1.38 (0.86–2.24)	0.186	1.14 (0.66–1.97)	0.632
Age	0.93 (0.91–0.96)	**< 0.001**	0.95 (0.92–0.99)	**0.008**
Time to surgery	0.96 (0.92–1.00)	0.071	0.96 (0.91–1.00)	0.075
Fracture type	2.87 (1.77–4.66)	**< 0.001**	2.89 (1.64–5.10)	**< 0.001**
Surgery duration	0.99 (0.99–1.00)	0.082	0.99 (0.99–1.00)	0.101
Blood loss	1.00 (1.00–1.00)	0.738	1.00 (1.00–1.00)	0.345
Blood transfusion	1.02 (0.51–2.01)	0.966	1.44 (0.57–3.64)	0.437
ASA	0.39 (0.20–0.76)	0.006	0.75 (0.36–1.58)	0.447
Anesthesia method	0.96 (0.36–2.56)	0.939	0.88 (0.29–2.70)	0.822
Leukocyte	1.12 (1.04–1.20)	**0.003**	1.11 (1.02–1.20)	**0.015**
Platelet	1.00 (1.00–1.00)	0.648	1.00 (1.00–1.00)	0.971
Hemoglobin	1.00 (0.98–1.02)	0.699	1.00 (0.98–1.03)	0.843
Creatinine	1.00 (1.00–1.01)	0.294	1.00 (1.00–1.01)	0.355
Albumin	0.97 (0.92–1.02)	0.204	0.98 (0.92–1.03)	0.4
Diabetes	1.00 (0.59–1.69)	0.993	1.07 (0.57–2.02)	0.833
Cardiovascular	0.61 (0.37–0.99)	0.048	0.72 (0.41–1.27)	0.260
Cerebrovascular	0.37 (0.23–0.58)	**< 0.001**	0.37 (0.22–0.65)	**< 0.001**
Chronic pulmonary	0.34 (0.22–0.54)	**< 0.001**	0.61 (0.35–1.05)	0.077
Renal insufficiency	0.34 (0.20–0.60)	**< 0.001**	0.51 (0.27–0.98)	**0.043**

OR, odds ratio; CI, confidence interval; ASA, American Society of Anesthesiologists; Bold values indicate statistical significance (*p* < 0.05).

### 3.4 Subgroup analyses

In the subgroup analyses, POD was treated as the dependent variable, and the nursing care model was treated as the exposure variable. After adjusting for nine covariates (gender, age, time to surgery, cerebrovascular disease, surgery duration, blood loss, blood transfusion, ASA classification, and anesthesia method), stratified subgroup analyses were conducted for all variables. Subgroup analyses based on age, gender, fracture type, time to surgery, surgery duration, blood loss, blood transfusion, anesthesia method, comorbidities, and laboratory indices revealed no significant interactions between the nursing care model and any covariate (all *P* for interaction > 0.05). The association between UNCM and POD risk remained consistent across all subgroups, indicating no evidence of effect modification. Subgroup analyses are shown in Supplementary Table 2.

## 4 Discussion

This study was conducted at a regional tertiary medical institution to investigate the association between UNCM and the incidence of POD in older adults with hip fractures. The results demonstrated that the incidence rate of POD was 15.29% in the UNCM group and 13.9% in the TNCM group, with no statistically significant difference between the groups. UNCM with restricted family visitation did not increase the occurrence of POD. This trend remained unchanged after adjusting for confounding factors.

Family participation can provide emotional support, orientation stimulation, feeding assistance, and other forms of care. It plays a key role in delirium prevention (11). A randomized clinical trial in older adults undergoing major non-cardiac surgery confirmed that patients receiving a tailored, family-involved hospital elder life program intervention had a significantly lower incidence of POD and a markedly shorter hospital stay (12). Qin et al. (13) reported that family participation intervention reduced the risk of delirium by 24% and was associated with fewer days of delirium. Another systematic review and network meta-analysis by Deng et al. (14) found that family participation was the most effective non-pharmacological intervention for reducing the incidence of delirium, followed by exercise programs, cerebral hemodynamic improvement, physical environment intervention, and sedation reduction. The underlying mechanisms may be as follows: Positive social support and family participation can downregulate hypothalamic–pituitary–adrenal axis activity, reduce cortisol release, and inhibit excessive inflammatory responses, thereby alleviating blood–brain barrier disruption and central nervous system inflammation, preserving neuronal function and network stability, and ultimately reducing the risk of POD (15, 16).

It remains unclear whether UNCM is associated with a higher incidence of POD due to reduced family participation. A retrospective observational study by Chen et al. (17) reported that UNCM, despite its advantages over standardized care services, was associated with a relatively high incidence of POD (25.36%) in older adults with hip fractures. This was the first study to report the incidence of POD under UNCM in this population. However, the main limitations of that study were the lack of a control group and the inherent limitations of an observational design, indicating that the causal relationship between UNCM and POD could not be confirmed. Our study addressed these limitations and confirmed that UNCM did not increase the incidence of POD in older adults with hip fractures, even with reduced family participation. Caregiver identity may be less important than the consistency, responsiveness, and quality of bedside care. However, this study was unable to obtain relevant information on the quality of nursing care.

One study found that interactive responses from caregivers, whether family members or medical staff, exert a significant impact on POD in patients undergoing cardiac surgery. Intimate care, respectful behavior, and effective communication can provide patients with a sense of security, whereas their absence may have the opposite effect (18). Another study demonstrated that psychoeducational interventions targeting caregivers can significantly improve their ability to manage delirium and reduce negative emotions (19). Education for family members can significantly reduce the incidence of delirium in ICU patients (20). These results collectively demonstrate that improving care quality is essential for preventing delirium. However, TNCM dominated by family members has certain limitations. Many family caregivers experience significant stress, anxiety, and burden during hospitalization (21, 22). These negative emotions may be transmitted to patients, potentially triggering delirium. Moreover, most family caregivers lack professional care skills and may be unable to manage postoperative pain, positioning, and complication monitoring. These deficiencies can be compensated for by professionally trained caregivers. Research has indicated that restricted visitation is associated with an increased incidence of POD (6, 7). To alleviate the adverse effects of restricted visitation, several studies have proposed using modern communication technologies, including telephone and video calls, to facilitate alternative communication between patients and their family members (23–25). Irrespective of the nursing care model, nurse-led, multidisciplinary, and multicomponent non-pharmacological intervention strategies are internationally recognized as the most effective measures for POD prevention (26–28). This provides a clear direction for optimizing delirium management strategies under UNCM.

## 5 Limitations

This study has several limitations. First, temporal variations caused by secular trends, policy updates, personnel changes, and COVID-19-related protocols may have affected the outcomes of this retrospective before-and-after cohort study. Second, POD was identified retrospectively based on medical records rather than a prospective systematic evaluation, leading to potential underdiagnosis, and misclassification that may have biased the comparative analyses. Third, several clinically important covariates related to delirium, such as pain, analgesia/sedation regimens, sedation depth, medications, frailty, functional dependence, anticholinergic drugs, sleep disruption, and care quality metrics, were not incorporated into the statistical model, introducing potential bias. Fourth, although PSM effectively balanced measurable baseline variables, substantial residual confounding from unrecorded and unquantifiable factors persisted.

## 6 Conclusion

UNCM may not increase the occurrence of POD in older adults with hip fractures and limited family participation.

## Data Availability

The datasets presented in this study can be found in online repositories. The names of the repository/repositories and accession number(s) can be found in the article/Supplementary material.
