# Supplementary material for: Association between the unaccompanied nursing care model and postoperative delirium in older adults with hip fractures: a retrospective before-and-after cohort study
Source: Front Med (Lausanne). 2026 Jul 6;13:1850834. doi: 10.3389/fmed.2026.1850834 (PMC13382537; doi:10.3389/fmed.2026.1850834)
Supplement: Supplementary file 1 [file Supplementary_file_1.doc]

**Supplementary Materials 1 Structured retrospective CAM-based chart review for POD**

| Inclusion criteria | Exclusion criteria |
| --- | --- |
| 1) The postoperative medical records contained "mental status change", "confusion", "disorientation", "agitation", "delirium", "inappropriate behaviour","inattention", "hallucinations", and "combative behaviour". | 1) Preoperative medical records containing the “symptoms” mentioned above |
| 2) The postoperative drug regimen contained "quetiapine", "olanzapine", "haloperidol", "haloperidol", and "risperidone". | 2) A preoperative drug regimen containing the “drugs” mentioned above |

POD was captured through descriptive words documented in the medical records and confirmed by JY and YL, who received standardized CAM training from psychiatrists. They rechecked all the delirium patients’ medical records, diagnosing the POD using Diagnostic and Statistical Manual of Mental Disorders, fifth edition (DSM-5) criteria.
