# Supplementary material for: Association between the unaccompanied nursing care model and postoperative delirium in older adults with hip fractures: a retrospective before-and-after cohort study
Source: Front Med (Lausanne). 2026 Jul 6;13:1850834. doi: 10.3389/fmed.2026.1850834 (PMC13382537; doi:10.3389/fmed.2026.1850834)
Supplement: Supplementary file 2 [file Table_1.docx]

# Supplementary table 1 Baseline characteristics and relative variables of the study population.

| **Variable** | **Unadjusted** | | | | | **Adjusted** | | | | |
| --- | --- | --- | --- | --- | --- | --- | --- | --- | --- | --- |
|  | **Total**  **(n = 645)** | **TNCM**  **(n = 403)** | **UNCM**  **(n = 242)** | ***P*** | **SMD** | **Total**  **(n = 478)** | **TNCM**  **(n = 239)** | **UNCM**  **(n = 239)** | ***P*** | **SMD** |
| Gender(female), n (%) | 458 (71.01) | 277 (68.73) | 181 (74.79) | 0.101 | 0.14 | 354 (74.06) | 176 (73.64) | 178 (74.48) | 0.835 | 0.019 |
| Age, M (Q₁, Q₃) | 82.00 (75.00, 88.00) | 82.00 (75.00, 87.00) | 82.00 (76.00, 88.00) | 0.727 | 0.04 | 82.00 (76.00, 88.00) | 83.00 (75.00, 88.00) | 82.00 (76.00, 88.00) | 0.901 | 0.022 |
| Time to surgery, M (Q₁, Q₃) | 5.00 (3.00, 7.00) | 5.00 (3.00, 7.00) | 5.00 (3.00, 7.00) | **0.038** | -0.114 | 5.00 (3.00, 7.00) | 5.00 (3.00, 7.00) | 5.00 (3.00, 7.00) | 0.707 | 0.023 |
| Fracture type, n (%) |  |  |  | 0.211 |  |  |  |  | 0.713 |  |
| Femoral neck | 311 (48.22) | 202 (50.12) | 109 (45.04) |  | -0.102 | 212 (44.35) | 104 (43.51) | 108 (45.19) |  | 0.034 |
| Intertrochanteric | 334 (51.78) | 201 (49.88) | 133 (54.96) |  | 0.102 | 266 (55.65) | 135 (56.49) | 131 (54.81) |  | -0.034 |
| Surgery duration, M (Q₁, Q₃) | 70.00 (58.00, 90.00) | 70.00 (60.00, 90.00) | 65.00 (55.00, 88.00) | **0.034** | -0.164 | 70.00 (55.00, 89.75) | 70.00 (58.50, 89.50) | 65.00 (55.00, 89.00) | 0.402 | -0.064 |
| Blood loss, M (Q₁, Q₃) | 200.00 (150.00, 300.00) | 250.00 (100.00, 350.00) | 200.00 (150.00, 300.00) | 0.545 | -0.051 | 200.00 (150.00, 300.00) | 250.00 (100.00, 300.00) | 200.00 (150.00, 300.00) | 0.839 | -0.017 |
| Blood transfusion, n (%) | 83 (12.87) | 56 (13.90) | 27 (11.16) | 0.315 | -0.087 | 51 (10.67) | 24 (10.04) | 27 (11.30) | 0.657 | 0.04 |
| ASA, n (%) |  |  |  | 0.576 |  |  |  |  | 0.524 |  |
| I/II | 160 (24.81) | 97 (24.07) | 63 (26.03) |  | 0.045 | 118 (24.69) | 56 (23.43) | 62 (25.94) |  | 0.057 |
| III/IV | 485 (75.19) | 306 (75.93) | 179 (73.97) |  | -0.045 | 360 (75.31) | 183 (76.57) | 177 (74.06) |  | -0.057 |
| Anesthesia method, n (%) |  |  |  | 0.076 |  |  |  |  | 0.648 |  |
| General | 601 (93.18) | 370 (91.81) | 231 (95.45) |  | 0.175 | 458 (95.82) | 230 (96.23) | 228 (95.40) |  | -0.04 |
| Regional | 44 (6.82) | 33 (8.19) | 11 (4.55) |  | -0.175 | 20 (4.18) | 9 (3.77) | 11 (4.60) |  | 0.04 |
| Leukocyte, M (Q₁, Q₃) | 10.80 (7.98, 12.01) | 10.79 (8.05, 11.89) | 10.84 (7.98, 12.10) | 0.754 | 0.008 | 10.87 (7.84, 12.01) | 10.89 (7.81, 11.89) | 10.79 (7.94, 12.05) | 0.986 | 0.001 |
| Platelet, M (Q₁, Q₃) | 243.00 (207.00, 281.00) | 238.00 (205.00, 280.50) | 247.00 (211.00, 284.75) | 0.163 | 0.114 | 246.00 (209.00, 284.00) | 245.00 (207.00, 284.00) | 247.00 (211.00, 284.50) | 0.646 | 0.028 |
| Hemoglobin, M (Q₁, Q₃) | 103.00 (97.00, 109.00) | 103.00 (97.00, 108.00) | 103.50 (97.25, 109.00) | 0.604 | 0.048 | 103.50 (98.00, 109.00) | 104.00 (99.00, 109.00) | 103.00 (97.00, 109.00) | 0.613 | -0.032 |
| Creatinine, M (Q₁, Q₃) | 89.00 (63.00, 112.00) | 90.00 (63.00, 113.50) | 88.00 (63.00, 109.00) | 0.661 | -0.042 | 90.00 (63.00, 113.00) | 94.00 (64.00, 116.00) | 88.00 (63.00, 109.00) | 0.277 | -0.075 |
| Albumin, M (Q₁, Q₃) | 36.00 (33.00, 38.00) | 36.00 (33.00, 38.00) | 36.00 (33.00, 38.00) | 0.784 | -0.04 | 36.00 (33.00, 38.00) | 36.00 (34.00, 38.00) | 36.00 (33.00, 38.00) | 0.798 | -0.058 |
| Diabetes, n (%) | 152 (23.57) | 92 (22.83) | 60 (24.79) | 0.569 | 0.045 | 119 (24.9) | 60 (25.10) | 59 (24.69) | 0.916 | -0.01 |
| Cardiovascular, n (%) | 397 (61.55) | 251 (62.28) | 146 (60.33) | 0.622 | -0.04 | 291 (60.88) | 145 (60.67) | 146 (61.09) | 0.925 | 0.009 |
| Cerebrovascular, n (%) | 243 (37.67) | 149 (36.97) | 94 (38.84) | 0.635 | 0.038 | 186 (38.91) | 95 (39.75) | 91 (38.08) | 0.707 | -0.034 |
| Chronic pulmonary, n (%) | 207 (32.09) | 137 (34.00) | 70 (28.93) | 0.182 | -0.112 | 141 (29.5) | 71 (29.71) | 70 (29.29) | 0.92 | -0.009 |
| Renal insufficiency, n (%) | 76 (11.78) | 45 (11.17) | 31 (12.81) | 0.531 | 0.049 | 65 (13.6) | 34 (14.23) | 31 (12.97) | 0.689 | -0.037 |
| TNCM, traditional-accompanied nursing care model; UNCM, unaccompanied nursing care model; SMD, standardized mean differences; ASA, american society of anesthesiologists; M(Q₁, Q₃), median (first quartile, third quartile) | | | | | | | | | | |
