# Supplementary material for: Association between the unaccompanied nursing care model and postoperative delirium in older adults with hip fractures: a retrospective before-and-after cohort study
Source: Front Med (Lausanne). 2026 Jul 6;13:1850834. doi: 10.3389/fmed.2026.1850834 (PMC13382537; doi:10.3389/fmed.2026.1850834)
Supplement: Supplementary file 3 [file Table_2.docx]

# Supplementary table 2 Interactions among nursing care model and each covariate.

| **Variables** | **n (%)** | **TNCM** | **UNCM** | **OR (95%CI)** | **P** | **P for interaction** |
| --- | --- | --- | --- | --- | --- | --- |
| All patients | 645 (100.00) | 56/403 | 37/242 | 1.17 (0.72 ~ 1.91) | 0.521 |  |
| Gender |  |  |  |  |  | 0.419 |
| Male | 187 (28.99) | 19/126 | 13/61 | 1.51 (0.63 ~ 3.65) | 0.358 |  |
| Female | 458 (71.01) | 37/277 | 24/181 | 0.96 (0.53 ~ 1.74) | 0.890 |  |
| Age, year |  |  |  |  |  | 0.456 |
| ＜82 | 310 (48.06) | 13/191 | 11/119 | 1.61 (0.66 ~ 3.90) | 0.292 |  |
| ≥82 | 335 (51.94) | 43/212 | 26/123 | 1.05 (0.58 ~ 1.88) | 0.882 |  |
| Time to surgery, day |  |  |  |  |  | 0.645 |
| ＜5 | 281 (43.57) | 23/161 | 16/120 | 1.06 (0.49 ~ 2.29) | 0.878 |  |
| ≥5 | 364 (56.43) | 33/242 | 21/122 | 1.29 (0.68 ~ 2.44) | 0.435 |  |
| Fracture type |  |  |  |  |  | 0.754 |
| Femoral neck | 311 (48.22) | 38/202 | 25/109 | 1.30 (0.68 ~ 2.49) | 0.435 |  |
| Intertrochanteric | 334 (51.78) | 18/201 | 12/133 | 1.03 (0.46 ~ 2.30) | 0.940 |  |
| Surgery duration, min |  |  |  |  |  | 0.418 |
| ＜70 | 300 (46.51) | 24/172 | 19/128 | 1.04 (0.51 ~ 2.09) | 0.923 |  |
| ≥70 | 345 (53.49) | 32/231 | 18/114 | 1.58 (0.78 ~ 3.20) | 0.207 |  |
| Blood loss, ml |  |  |  |  |  | 0.335 |
| ＜200 | 244 (37.83) | 15/150 | 15/94 | 1.71 (0.73 ~ 4.02) | 0.220 |  |
| ≥200 | 401 (62.17) | 41/253 | 22/148 | 1.00 (0.55 ~ 1.85) | 0.988 |  |
| Blood transfusion |  |  |  |  |  | 0.798 |
| No | 562 (87.13) | 47/347 | 32/215 | 1.16 (0.68 ~ 1.99) | 0.580 |  |
| Yes | 83 (12.87) | 9/56 | 5/27 | 1.45 (0.37 ~ 5.71) | 0.597 |  |
| ASA |  |  |  |  |  | 0.967 |
| I/II | 160 (24.81) | 7/97 | 4/63 | 1.28 (0.30 ~ 5.44) | 0.742 |  |
| III/IV | 485 (75.19) | 49/306 | 33/179 | 1.19 (0.71 ~ 2.00) | 0.511 |  |
| Anesthesia method |  |  |  |  |  | 0.790 |
| General | 601 (93.18) | 51/370 | 35/231 | 1.22 (0.74 ~ 2.02) | 0.437 |  |
| Regional | 44 (6.82) | 5/33 | 2/11 | 0.54 (0.02 ~ 12.29) | 0.700 |  |
| Leukocyte, ×10^9^/L |  |  |  |  |  | 0.667 |
| ＜10.80 | 322 (49.92) | 34/202 | 22/120 | 1.09 (0.57 ~ 2.08) | 0.797 |  |
| ≥10.80 | 323 (50.08) | 22/201 | 15/122 | 1.31 (0.62 ~ 2.78) | 0.477 |  |
| Platelet, ×10^9^/L |  |  |  |  |  | 0.907 |
| ＜243 | 320 (49.61) | 29/208 | 16/112 | 1.21 (0.56 ~ 2.65) | 0.629 |  |
| ≥243 | 325 (50.39) | 27/195 | 21/130 | 1.19 (0.62 ~ 2.29) | 0.609 |  |
| Hemoglobin, g/L |  |  |  |  |  | 0.804 |
| ＜103 | 298 (46.20) | 23/186 | 16/112 | 1.25 (0.59 ~ 2.65) | 0.555 |  |
| ≥103 | 347 (53.80) | 33/217 | 21/130 | 1.12 (0.58 ~ 2.17) | 0.735 |  |
| Creatinine, umol/L |  |  |  |  |  | 0.606 |
| ＜89 | 318 (49.30) | 29/196 | 17/122 | 1.12 (0.54 ~ 2.33) | 0.759 |  |
| ≥89 | 327 (50.70) | 27/207 | 20/120 | 1.28 (0.66 ~ 2.51) | 0.465 |  |
| Albumin, g/L |  |  |  |  |  | 0.307 |
| ＜36 | 289 (44.81) | 29/182 | 15/107 | 0.85 (0.41 ~ 1.74) | 0.648 |  |
| ≥36 | 356 (55.19) | 27/221 | 22/135 | 1.53 (0.77 ~ 3.03) | 0.226 |  |
| Diabetes |  |  |  |  |  | 0.967 |
| No | 493 (76.43) | 41/311 | 28/182 | 1.16 (0.66 ~ 2.04) | 0.612 |  |
| Yes | 152 (23.57) | 15/92 | 9/60 | 1.29 (0.47 ~ 3.57) | 0.622 |  |
| Cardiovascular |  |  |  |  |  | 0.200 |
| No | 248 (38.45) | 13/152 | 13/96 | 1.86 (0.77 ~ 4.52) | 0.169 |  |
| Yes | 397 (61.55) | 43/251 | 24/146 | 0.97 (0.53 ~ 1.77) | 0.925 |  |
| Cerebrovascular |  |  |  |  |  | 0.909 |
| No | 402 (62.33) | 24/254 | 14/148 | 1.30 (0.61 ~ 2.79) | 0.493 |  |
| Yes | 243 (37.67) | 32/149 | 23/94 | 1.17 (0.61 ~ 2.23) | 0.639 |  |
| Chronic pulmonary |  |  |  |  |  | 0.163 |
| No | 438 (67.91) | 23/266 | 22/172 | 1.59 (0.82 ~ 3.08) | 0.165 |  |
| Yes | 207 (32.09) | 33/137 | 15/70 | 0.84 (0.39 ~ 1.79) | 0.649 |  |
| Renal insufficiency |  |  |  |  |  | 0.753 |
| No | 569 (88.22) | 43/358 | 28/211 | 1.16 (0.68 ~ 1.99) | 0.581 |  |
| Yes | 76 (11.78) | 13/45 | 9/31 | 1.20 (0.29 ~ 5.02) | 0.801 |  |
| TNCM, traditional-accompanied nursing care model; UNCM, unaccompanied nursing care model; ASA, american society of anesthesiologists | | | | | | |
